# Molecular dynamic simulations to assess the structural variability of ClpV from *Enterobacter cloacae*

**DOI:** 10.3389/fbinf.2025.1498916

**Published:** 2025-03-25

**Authors:** Tehrim Motiwala, Babalwa Nyide, Thandeka Khoza

**Affiliations:** Department of Biochemistry, School of Life Sciences, Pietermaritzburg Campus, University of KwaZulu-Natal, Pietermaritzburg, South Africa

**Keywords:** *Enterobacter*, *Enterobacter cloacae*, Clp ATPases, ClpV, antibiotic resistance, alternative therapy

## Abstract

The *Enterobacter cloacae* complex (ECC) consists of six *Enterobacter* species (*E. cloacae*, *hormaechei*, *kobei*, *ludwigii*, *nimipressuralis* and *asburiae*) that have emerged as nosocomial pathogens of interest, with *E. cloacae* and *Enterobacter hormachei* being the most frequently isolated ECC species in human clinical specimens and intensive care unit (ICU) patients. Many nosocomial outbreaks of *E. cloacae* have been related to transmission through contaminated surgical equipment and operative cleaning solutions. As this pathogen evades the action of antibiotics, it is important to find alternative targets to limit the devastating effects of these pathogens. ClpV is a Clp ATPase which dissociates and recycles the contracted sheath of the bacterial type VI secretion system (T6SS), thereby regulating bacterial populations and facilitating environmental colonization. Seventy-one *Enterobacter* strains were mined for Clp ATPase proteins. All the investigated strains contained ClpA, ClpB, ClpX and ClpV while only 20% contained ClpK. All the investigated strains contained more than one ClpV protein, and the ClpV proteins showed significant variations. Three ClpV proteins from *E. cloacae* strain E3442 were then investigated to determine the structural difference between each protein. Homology modelling showed the proteins to be structurally similar to each other, however the physicochemical characteristics of the proteins vary. Additionally, physicochemical analysis and molecular dynamic simulations showed that the proteins were highly dynamic and not significantly different from each other. Further investigation of the proteins *in silico* and *in vitro* in the presence and absence of various ligands and proteins could be performed to determine whether the proteins all interact with their surroundings in the same manner. This would allow one to determine why multiple homologs of the same protein are expressed by pathogens.

## 1 Introduction

*Enterobacter* spp. are a genus of Gram-negative bacteria belonging to the Enterobacteriaceae family. Microorganisms of this genus form part of the commensal microflora of the gastrointestinal (GI) tract of humans and animals (Davin-Regli et al., 2019; Mezzatesta et al., 2012). In more recent years, the *Enterobacter* spp. have become subject to increasing clinical scrutiny and are recognized as one of the bacterial pathogens described as ESKAPE pathogens which evade the effects of many different classes of antibiotics (Davin-Regli et al., 2019; De Oliveira D. M. et al., 2020; Pendleton et al., 2013; De Oliveira D. M. P. et al., 2020).

Furthermore, the *Enterobacter* spp. have been identified as the causative agents of post-surgical peritonitis, hospital-acquired sepsis, nosocomial urinary tract infections, neonatal pneumonia cases, bloodstream infections, endocarditis, septic arthritis, and intra-abdominal infection and therefore emerged as nosocomial pathogens of interest (De Oliveira D. M. et al., 2020; Mezzatesta et al., 2012; Ghazvini and Farsiani, 2020; Davin-Regli et al., 2019). From the six *Enterobacter* spp. of the *Enterobacter cloacae* complex (ECC), *E. cloacae* and *Enterobacter hormachei* are the most frequently isolated ECC species in human clinical specimens and intensive care unit (ICU) patients. *E. cloacae* outbreaks have been related to transmission through contaminated surgical equipment and operative cleaning solutions. This may be due to their ability to colonize medical devices and intravenous hospital equipment among other objects. More interestingly, there is evidence to suggest that healthcare workers can act as reservoirs for transmission of *E. cloacae* given that this bacterium is commonly contracted through the skin (Van Nierop et al., 1998; Mezzatesta et al., 2012).

*E. cloacae* are resilient, dynamic, and opportunistic pathogens with the ability to upregulate resistance determinants or evolve and adapt to overcome arising challenges in the environment. Their diverse arsenal of strategies includes (but is not limited to) the acquisition of resistance-conferring genes through horizontal gene transfer, and the ability to overproduce AmpC β-lactamases by mutation or depression of a chromosomal gene (Davin-Regli et al., 2019; Huang et al., 2012; Jacoby, 2009; Kaneko et al., 2005). Moreover, through the action of the Bush group 1 inducible natural cephalosporinase, *E. cloacae* are intrinsically resistance to various antimicrobial compounds including amoxicillin, amoxicillin-clavulanic acid, ampicillin, cefoxitin and cephalothin (Davin-Regli and Pagès, 2015; Mezzatesta et al., 2012). There is a growing body of evidence that links caseinolytic proteins (Clp) and pathogenicity of ESKAPE pathogens and this has unearthed exciting new avenues for anti-microbial drug discovery efforts targeting Clp proteins (D’andrea et al., 2022; Dhiman and Singh, 2018; Motiwala et al., 2022).

Clp proteins belong to the AAA+ (ATPases associated with diverse cellular activities) superfamily that facilitate protein unfolding, refolding, and tolerance to heat stress and oxidative stress (Bouchnak and Van Wijk, 2021; Sen et al., 2020; Voos, 2013). Clp proteins consist of the peptidase (ClpP) and the regulatory (Clp ATPase). Clp ATPases are characterized by their distinct nucleotide-stabilized ring-shaped multimeric structures (Neuwald et al., 1999; Bouchnak and Van Wijk, 2021). Clp ATPases are classified based on the number of nucleotide binding domains (NBDs) they possess; Class I members (Clps A, B, C, D, E, K, L and V) contain two NBDs, whereas Class II members (Clps M, N, X and Y) contain only one (Motiwala et al., 2022; Schlieker et al., 2005). The NBD is characterized by a conserved sequence of between 200–250 amino acids that make up the Walker A and Walker B motifs, which are responsible for ATP binding and hydrolysis (Bouchnak and Van Wijk, 2021; Schlieker et al., 2005; Seraphim and Houry, 2020).

To execute their functions, Clp ATPases can operate via two mechanisms that depend on the presence or absence of the tripeptide consensus sequence (IGF/L) on a helix-loop-helix motif of the NBD (Kim et al., 2001; Schlieker et al., 2005). Clp ATPases of the ClpA sub-family commonly possess this tripeptide, which is required for interaction with the Clp peptidase, ClpP (Kim et al., 2001; Schlieker et al., 2005). These ATPases recruit, refold, and reactivate misfolded proteins. Alternatively, they recruit misfolded proteins and then bind to inactive ClpP, a serine protease comprising two heptameric rings, thereby triggering a conformational change that realigns the residues of the catalytic triad and activates the protease site of ClpP (Brötz-Oesterhelt and Sass, 2014; Capestany et al., 2008). Proteins that are unsuccessfully reactivated are degraded through this proteolytic complex, which comprises of one ClpP tetradecamer sandwiched between two regulatory ATPase hexamers (Frees et al., 2007; Kirstein et al., 2009; Schlieker et al., 2005). By contrast, Clp ATPases of the ClpB/Hsp104 sub-family, which lack this tripeptide, can resolubilize and reactivate misfolded proteins alone or through cooperation with chaperones of the DnaK/DnaJ/GrpE system (Motiwala et al., 2022; Schlieker et al., 2005; Wickner et al., 1994; Zavilgelsky et al., 2004; Zolkiewski, 1999).

ClpV dissociates and recycles the contracted sheath of the bacterial type VI secretion system (T6SS) for new injection cycles (Förster et al., 2014; Douzi et al., 2016). T6SS is an offensive system used by bacteria to compete with environmental rivals and is responsible for the transport and delivery of toxins to target cells, thus regulating bacterial populations (Douzi et al., 2016; Smith-Roberge et al., 2018). Overall, members of the ClpB/Hsp104 and ClpV sub-families share approximately 30%–35% primary sequence identity (Schlieker et al., 2005). Like the ClpB/Hsp104 sub-family, homologs of the ClpV sub-family lack the IGF/L tripeptide sequence, suggesting that ClpV ATPases function independently of ClpP and the substrate proteolysis mechanism (Schlieker et al., 2005). The widespread distribution of Clp proteins across many microbial genera is a testament to their importance. Evidently, the role of maintaining protein homeostasis is crucial for cell survival, making a strong case for the targeting of Clp proteins for antimicrobial drug studies (Brötz-Oesterhelt and Sass, 2014; Frees et al., 2007). Here, we show the distribution of Clp proteins among strains of the *Enterobacter* genus, and the phylogenetic analysis of ClpV, the dominant Clp ATPase within the clinically significant *E. cloacae* species.

## 2 Material and methods

### 2.1 Species and databases

The National Center for Biotechnology Information (NCBI) Genome database (https://www.ncbi.nlm.nih.gov/home/genomes/) was browsed by organism to collect protein genomes of 71 *Enterobacter* strains (17 species, complete draft). The 71 *Enterobacter* strains which were investigated included; 27 *Enterobacter hormaechei* strains, 1 *Enterobacter cancerogenus* strain, 6 *Enterobacter ludwigii* strains, 6 *Enterobacter roggenkampii* strains, 2 *Enterobacter bugandensis* strains, 13 *E. cloacae* strains, 4 *Enterobacter asburiae* strains, 3 *Enterobacter kobei* strains, 1 *Enterobacter chengduensis* strain, 1 *Enterobacter sichuanensis* strain, 1 *Enterobacter oligotrophicus* strain, 1 *E. N18-03635* strain, 1 *E. JBIWA003* strain, 1 *E. JBIWA005* strain, 1 *E. JBIWA008* strain, 1 *E. DNB-S2* strain and 1 *E. SGAir0187* strain.

### 2.2 Genome data mining and Clp ATPase annotation

Clp ATPases were mined from different *Enterobacter* strains by first obtaining the protein file for each strain from NCBI. Each file was then converted from.gz to FASTA format using an online server (https://www.convertfiles.com/file_type_description/GZ_Archive_FIle.html). The FASTA files were then individually searched for the presence of Clp ATPases. The Clp ATPase sequences were separated from the main file and used for further analyses.

### 2.3 Phylogenetic analysis

The Clp ATPase protein sequences were aligned using MAFFT v6.864 embedded on the T-rex web server (http://www.trex.uqam.ca/) (Katoh et al., 2005; Boc et al., 2012). The alignments were deduced by the T-rex server, following this the file for the best tree was downloaded, visualized and colored using the interactive Tree of Life (iTOL) v6 server (https://itol.embl.de/) (Letunic and Bork, 2021). The phylogenetic tree (Neighbor-joining, BLOSUM62) of ClpV in comparison with ClpVI from *Pseudomonas aeruginosa* (UniProt: Q9I742) and *Vibrio cholera* ClpV1 (UniProt: A0A395TZY1) was constructed using JalView v 2.11.2.2 (Waterhouse et al., 2009a).

### 2.4 Clp ATPase homology analysis

Clustal omega (https://www.ebi.ac.uk/jdispatcher/msa/clustalo) was used in four ways during the investigation. Firstly, the percentage identity between the various Clp ATPase classes was analyzed. Secondly, the percentage identity between the ClpV mined from the *E. cloacae* strains, *P. aeruginosa* ClpV1 (UniProt: Q9I742), and *V. cholera* ClpV1 (UniProt: A0A395TZY1) was compared. Thirdly, the percentage identity between the *E. cloacae* E3442 ClpV homologs were analyzed. Lastly, the percentage identity between the three E3442 ClpV proteins and *V. cholera* ClpV1 (UniProt: A0A395TZY1) was investigated (Sievers et al., 2011). The domains were assigned to the ClpV proteins using the annotations provided through InterPro (Blum et al., 2020).

### 2.5 ClpV homology modelling

The three ClpV proteins mined from *E. cloacae* E3342 were modelled using the template 1qvrA through Swiss Model (https://swissmodel.expasy.org/) (Waterhouse et al., 2018). The structures were refined using the Protein REFinement via Molecular Dynamics (PREFMD) web server (https://feig.bch.msu.edu/prefmd) (Heo and Feig, 2017). Structural assessment of the refined structures was then performed using the Swiss-Model assessment tool (https://swissmodel.expasy.org/assess) and MolProbity (http://molprobity.biochem.duke.edu/) (Waterhouse et al., 2018; Williams et al., 2018). The refined structures were superimposed with the modelling template (PDB 1qvrA) using PyMol (Schrödinger, 2020).

### 2.6 ClpV comparison

The ProtParam tool on the Expasy server (https://web.expasy.org/protparam/) was used to compare the physicochemical properties of the three ClpV proteins using the respective protein sequences (Gasteiger et al., 2003). ClpV protein disorder was predicted using the IUPred3 server (https://iupred3.elte.hu/) (Erdős et al., 2021). ClpV sequences were analyzed using Mafft on the Jalview software and the InterPro server (http://www.ebi.ac.uk/interpro/) was used to investigate the protein families, domains and function of the ClpV sequences (Waterhouse et al., 2009b; Apweiler et al., 2001). Normal Mode Analysis (NMA) from the DynaMut server (https://biosig.lab.uq.edu.au/dynamut/analysis) was performed to compare the conformation of the ClpV proteins (Rodrigues et al., 2018). The ClpV sequences were annotated as per the InterPro server and Lee et al. (2003). The 3D structures of the ClpV proteins were then compared using the Dali server (http://ekhidna2.biocenter.helsinki.fi/dali/) (Holm, 2022). Each ClpV protein sequence was analyzed using the STRING v11.5 server (https://string-db.org/) for protein-protein association analysis (Szklarczyk et al., 2019). The minimum required interaction score was set to medium (0.400). DogSite3 was used through the ProteinsPlus server (https://proteins.plus/) to predict binding sites on the ClpV proteins (Graef et al., 2023; Schöning-Stierand et al., 2022; Schöning-Stierand et al., 2020; Fährrolfes et al., 2017).

### 2.7 Molecular simulations and post dynamic studies

Molecular dynamic (MD) simulations were performed using Maestro v12.2 through the implemented GPU-enabled Desmond molecular dynamics simulation engine (Schrodinger, 2006). The modelled ClpV structures were saved as Protein Data Bank (PDB) files and submitted to the Linux (Ubuntu) desktop server for molecular simulations studies. The system builder module was used to incorporate the TIP3P solvent model with the OPLS forcefield. The proteins were placed in an orthorhombic box (distance from the box face to the outermost protein atom was set to 10 Å, the box angle was α = β = γ = 90°). The volume box containing the proteins was minimized, and counter ions were added to neutralize the system. In addition, 0.15 M NaCl was added into the solvent box for physiological conditioning. The system was then submitted for MD simulations for 250 ns.

Post dynamic analyses of the trajectories derived from the MD simulation studies were performed using Schrodinger Maestro v12.2. Firstly, Simulation Quality Analysis was used to analyze the quality of simulations through the average energy, pressure, temperature, and volume analysis. Secondly, Simulation Interaction Diagram algorithm was used to analyze the RMSD of the alpha carbon atoms (Cα), the root mean square fluctuation (RMSF) of the residues, and secondary structure element analysis. Lastly, the Simulation Events Analysis algorithm (implemented in Maestro v12.2) was used to calculate the radius of gyration (Rg).

## 3 Results

### 3.1 Clp ATPase classification and phylogenetic analysis

Advancement into sequencing technologies enables researchers to study the presence and distribution of genes in microorganisms to shed light into relationships amongst species (Pareek et al., 2011). Clp ATPases are essential for bacterial survival and pathogenicity and are emerging as potential drug targets (Culp and Wright, 2017). However, it was observed that the presence and distribution of Clp ATPases in the *Enterobacter* species has not been fully investigated hence, genome wide mining was performed to gain an insight into the diversity of Clp ATPases in the *Enterobacter* species.

**FIGURE 1:**
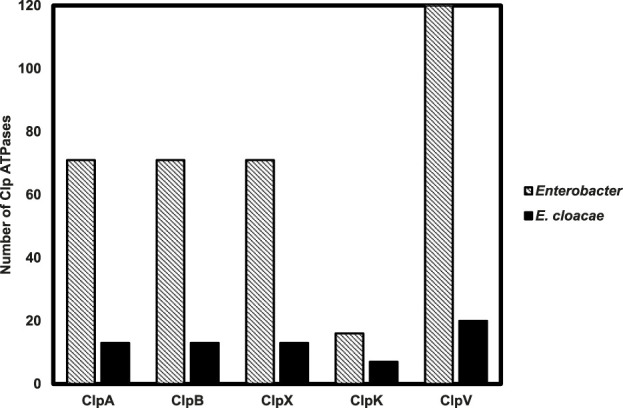
Distribution of Clp ATPases across 71 *Enterobacter* strains with a focus on the Clp ATPases identified in 13 *Enterobacter cloacae* strains. The number of Clp ATPases mined from the 71 *Enterobacter* strains and 13 *Enterobacter cloacae* strains through the NCBI genome database were tallied.

The data mined and tallied for the 71 *Enterobacter* species shows that all the studied species have both Class I (ClpA and ClpB) and Class II (ClpX and ClpV) Clp ATPase members (Figure 1). It was also observed that fewer *Enterobacter* species (20%) contained ClpK, a class II member of Clp ATPases that has been recently linked to thermotolerance properties of another ESKAPE pathogen member, *Klebsiella pneumonia* (Bojer et al., 2010; Motiwala et al., 2021). It was very interesting to observe from genome data mining that all investigated strains contained one gene copy for ClpA, ClpB and ClpX. Contrastingly, certain *Enterobacter* strains contained more than one copy of the ClpV gene.

**FIGURE 2:**
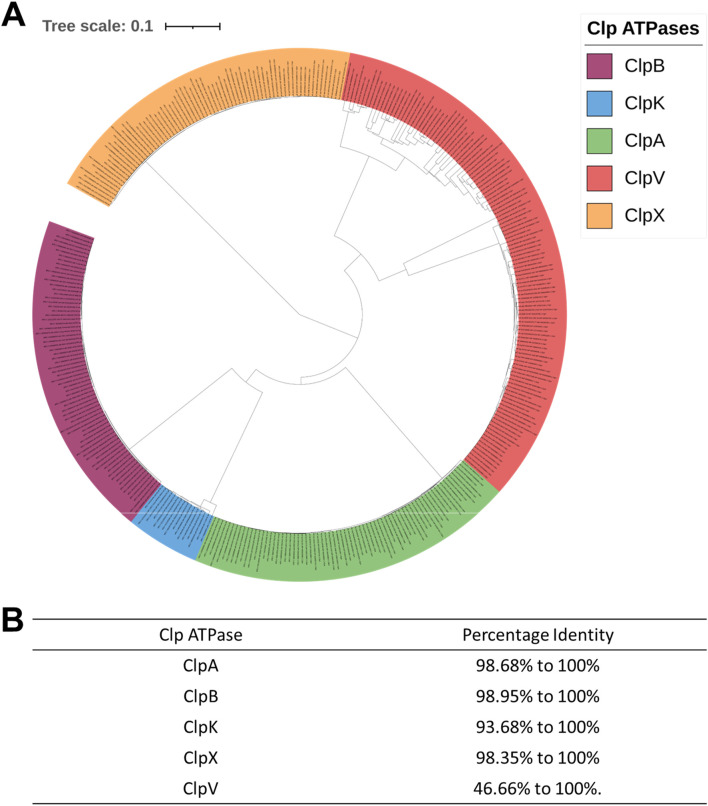
Phylogenetic tree and Clustal analysis of Clp ATPases mined from 71 *Enterobacter* strains. **(A)** Phylogenetic tree of the mined Clp ATPases. The alignment for the tree was done using MAFFT embedded on the Trex servers. The tree was visualized and colored using iTOL. The various Clp ATPases are represented in assorted colors. Tree distance scale: 0.1. **(B)** Clustal analysis of the Clp ATPases mined from the *Enterobacter* strains.

It then became necessary to ascertain the evolutionary relationship of the Clp ATPase species in *Enterobacter* species. Subsequently, we constructed a phylogenetic tree of the mined Clp ATPases. Figure 2 showed that the ClpA, ClpB, ClpK and ClpV proteins diverge from one point. This is expected as all these proteins contain two nucleotide domains and belong to Clp ATPase Class I. Therefore, one would assume that they would be more closely related to each other than to ClpX which belongs to Clp ATPase Class II (Figure 2A). Analysis of the phylogenetic tree shows that there is not a great amount of genetic variation among the ClpA, ClpB, ClpX and ClpK proteins, however, there appears to be significant genetic variation among the ClpV proteins (Figure 2A). This correlates with Clustal omega analysis of each protein group (Figure 2B).

**FIGURE 3:**
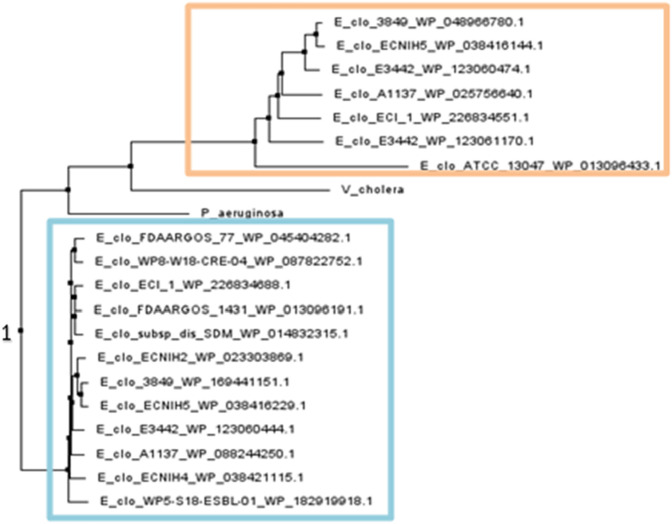
Neighbor Joining tree of ClpV mined from *Enterobacter cloacae* in comparison to ClpVI from *Pseudomonas aeruginosa* and *Vibrio cholera*. The tree was constructed using the Neighbor Joining method (BLOSUM62) on Jalview v2.11.2.2 (Waterhouse et al., 2009a). The protein codes for each mined ClpV protein are displayed. The orange box outlines one group of ClpV proteins while the blue box outlines the second group. The common point of origin of the ClpV proteins is indicated by the number 1.

The role of ClpV proteins has been widely studied in Gram-negative opportunistic pathogens; *V. cholera* and *P. aeruginosa* (Smith-Roberge et al., 2018; Sun et al., 2014; Lesic et al., 2009; Li et al., 2020). The ClpV proteins mined from various *E. cloacae* strains were compared to ClpV, from *V*. *cholera* and *P*. *aeruginosa* through Clustal omega and phylogenetic analysis. This was done to elucidate whether the ClpV proteins from the *E. cloacae* strains may have a similar function to those of ClpV1 from *V*. *cholera* and *P*. *aeruginosa.* Clustal omega showed that ClpV from the various *E. cloacae* strains had between 43.56% and 100% similarity with ClpV1 from *P. aeruginosa*, and between 43.31% and 100% similarity with ClpV1 from *V. cholera*. ClpV from *E. cloacae* had a percentage identity of greater than 30% in comparison to ClpV1 from both *V. cholera* and *P. aeruginosa*, therefore indicating that the sequences are homologous (Pearson, 2013). Additionally, analysis of the phylogenetic tree comparing the evolution of ClpV from *E. cloacae* and ClpV1 from both *V. cholera* and *P. aeruginosa* showed that these proteins all shared a common ancestor, indicated by the number 1 (Figure 3). Figure 3 shows that a group of ClpV from *E. cloacae* (orange box) are more closely related to ClpVI from *V*. *cholera* and *P*. *aeruginosa* compared to the other group of ClpV from *E. cloacae* (blue box). Clustal omega analysis showed that three mined ClpV proteins from the E3442 strain had between 46.82% and 100% similarity, this correlated with the trend observed in Figure 3 which shows that these proteins lie on different branches of the phylogenetic tree.

### 3.2 Homology modelling of ClpV

**FIGURE 4:**
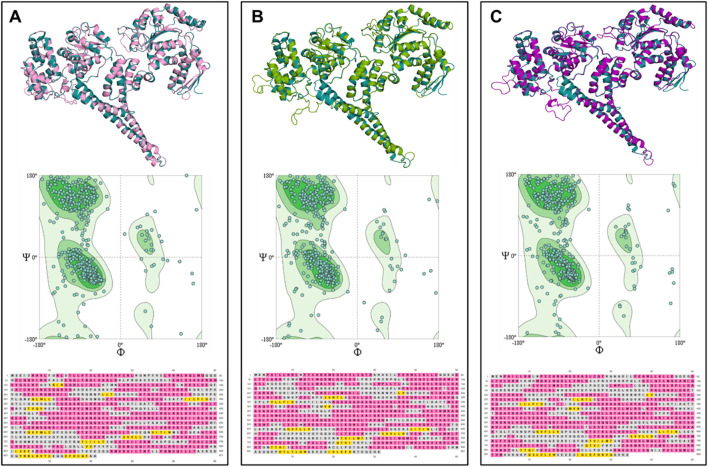
Tertiary structure, Ramachandran plot and secondary structure analysis of three ClpV proteins from *E. cloacae strain* E3442. **(A)** ClpV1 (lilac) and 1qvrA template (blue). **(B)** ClpV2 (green) and 1qvrA template (blue). **(C)** ClpV3 (purple) and 1qvrA template (blue). The α-helical residues are colored pink and the β-sheets and yellow.

To further investigate the uniqueness of the ClpV proteins identified within *E. cloacae* strain E3442, the three ClpV proteins were compared through homology modelling. The ClpV proteins were modelled using the 1qvrA template and the model quality was assessed (Figure 4; Table 1). The alignment of ClpV1, ClpV2, and ClpV3 with the template (1QVR-A) revealed a root mean square deviation (RMSD) score of 0.270, 0.283 and 0.347, respectively. These scores are close to 0 and indicate that there is a good alignment between the coordinates of the model and template. Therefore, the proteins are structurally similar to the template (Carugo and Pongor, 2001). Secondary and tertiary analysis of the proteins show that they are mainly α-helical (Figure 4).

**TABLE 1:** Assessment of the model quality of the modelled ClpV proteins from *Enterobacter cloacae strain* E3442.

	Rama Z-score	Ramachandran favored	QMeanisCo
ClpV1	−1.37 ± 0.28	88.89%	0.58 ± 0.05
ClpV2	−1.52 ± 0.28	88.39%	0.60 ± 0.05
ClpV3	−1.05 ± 0.28	88.40%	0.60 ± 0.05

The Rama Z-score provides an overall insight into the quality of a modelled protein by comparing the model to a reference set of high-resolution structures (Sobolev et al., 2020). The Rama Z-scores of the modelled proteins were less than two and therefore the modelled structures are confirmed to be of adequate quality (Table 1). The Ramachandran favored scores of the three modelled proteins are greater than 80%, this indicates that the generated models can be trusted and used for further analysis (Maxwell and Popelier, 2017). The QMeanisCo uses statistical potentials of mean force to quantify model quality and modelling errors (Studer et al., 2019). The QMeanisCo values for the three modelled proteins are close to 1, thus once again confirming that the models are of adequate quality (Table 1).

### 3.3 Comparing ClpV

**FIGURE 5:**
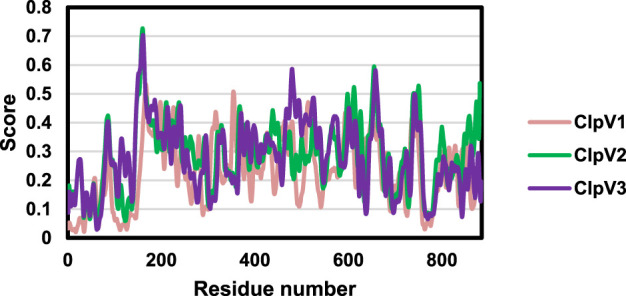
Disorder prediction of the three ClpV proteins. The disorder of ClpV1 (pink), ClpV2 (green), and ClpV3 (purple) proteins was predicted using the IUPred3 server.

Table 2 shows the physicochemical properties of the three ClpV proteins. The instability index of all three proteins is greater than 40, the proteins are therefore considered to be unstable. However, it is essential to remember that the stability of the protein is not only determined by the intrinsic nature of the protein but also by the protein environment (Gamage et al., 2019). The high aliphatic indexes indicate that these proteins are thermally stable. Additionally, the negative Grand average of Hydropathicity (GRAVY) score indicates that the proteins are all hydrophilic (Panda and Chandra, 2012). Figure 5 shows the protein disorder prediction, the disorder score for the residues of all three proteins lie below one and the three proteins are not considered to be disordered. Therefore, these proteins can be expressed and purified (Deng et al., 2012).

**TABLE 2:** Comparison of the physicochemical properties of the three ClpV proteins.

	ClpV1	ClpV2	ClpV3
Molecular Weight (kDa)	95.50	97.66	98
Instability Index	45.52	42.58	45.52
Aliphatic Index	103.08	101.24	101.55
GRAVY^a^ score	−0.097	−0.240	−0.254

^a^ Grand Average of Hydropathicity.

**FIGURE 6:**
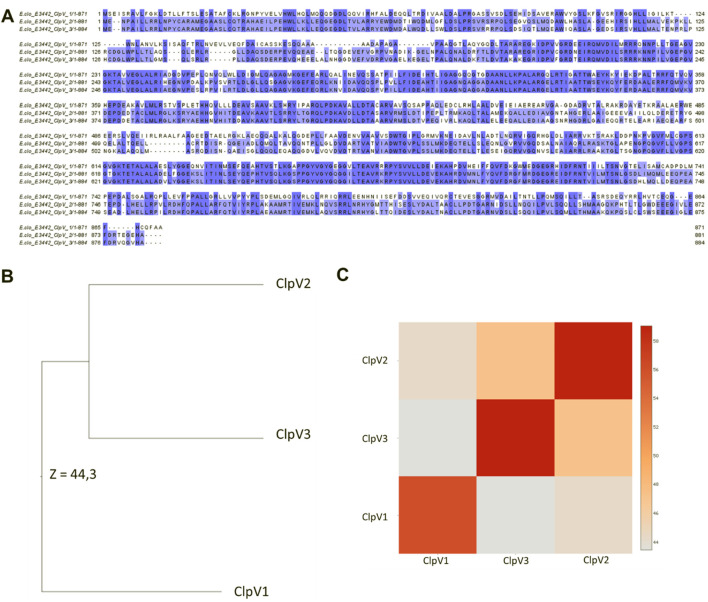
Comparison of the sequence and 3D structures of the three ClpV proteins using the Jalview software and Dali server, respectively. **(A)** Multisequence alignment performed using Mafft on the Jalview software. Alignment colored according to percentage identity. **(B)** Average linkage clustering of the Dali Z-scores is used to construct a Dendrogram. **(C)** Dali Z-scores are used to construct a heatmap.

Following analysis of the physicochemical characteristics and disorder prediction of the proteins, the difference in the protein sequence and 3D structure were further investigated. Figure 6A shows significant sequence similarity and conservation, especially between ClpV2 and ClpV3. The difference observed between the protein sequences indicate that these proteins may have variable enzyme activity and binding affinities. The 3D structure analysis corresponds with the primary sequence analysis in that ClpV2 and ClpV3 fall on the same branch of the phylogenetic tree (Figures 6B,C). Additionally, the Dali Z-score of 44.3 is greater than 20 and confirms that the three ClpV structures are homologous to each other (Holm et al., 2008).

**FIGURE 7:**
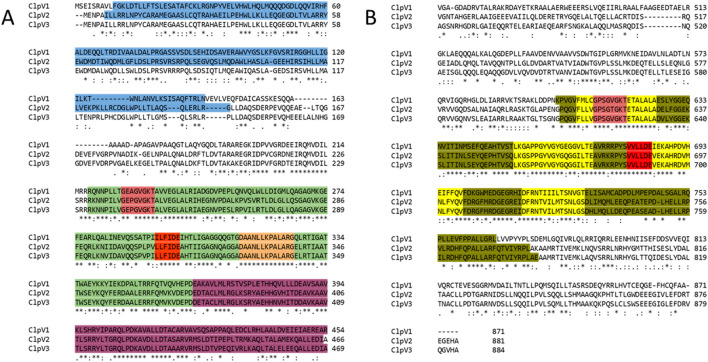
Alignment of the three ClpV proteins. The annotations were done according to the InterPro server and Lee et al. (2003). Asterisks (*), colons (:) and full stops (.) represent the identical residues, sequence homologies, and weak similarities, respectively. The residues are highlighted blue, green, pink, red, orange, purple, olive and yellow. These represent the repeat domain, Nucleotide Binding Domain (NBD) 1, Walker A motif, Walker B motif, conserved site 1, AAA lid domain, NBD II, and conserved regions of the Clp A/B family. **(A)** First half of the alignment (residues 1 to 496). **(B)** Second half of alignment.

The InterPro server analysis identified protein families, domains, and functional sites in ClpV proteins (Apweiler et al., 2001). ClpV1 and ClpV2 contain the repeat domain, which plays a key role in substrate specificity, a characteristic feature of the Clp A/B family (Lo et al., 2001). The nucleotide binding domains (NDB I and II) which are crucial for ATP binding and are well conserved among Clp A/B family members (Gottesman et al., 1990). They exhibit high sequence identity, with only minor variations across the three ClpV proteins. A highly conserved site (highlighted in orange) is situated in NBDI and remains identical across the three ClpV proteins (Figure 7). Similar to ClpB and ClpK, ClpV proteins contain a middle linker region that separates the NBDs and is essential for chaperone activity (Lee et al., 2003; Motiwala et al., 2021). Additionally, the AAA + lid domain, located in the middle domain of ClpV proteins, acts as a regulatory “gatekeeper” for substrate access through the central pore (Miller and Enemark, 2016).

Further conserved regions include the Walker A (highlighted in pink) and Walker B (highlighted in red) motifs, located within NBD I and NBD II. The Walker A motif, which is responsible for ATP binding, is largely conserved, though a single amino acid variation suggests potential differences in ATP interactions among the three proteins. The Walker B motif, which facilitates metal ion binding, remains identical and homologous across ClpV proteins, indicating a shared metal ion interaction mechanism (Lee et al., 2003; Motiwala et al., 2021). Conserved residues (highlighted in yellow) further support the structural and functional similarity of the ClpV proteins (Figure 7).

**FIGURE 8:**
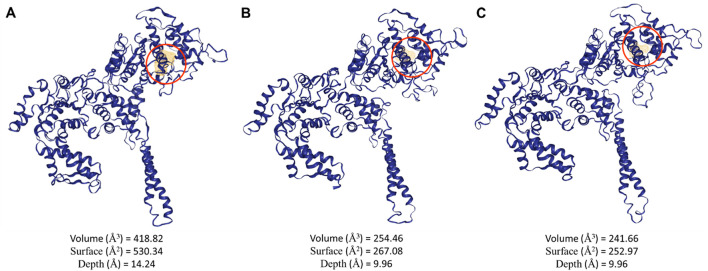
Potential binding pockets of the three ClpV proteins identified using the DogSite3 module on the ProteinsPlus server. **(A)** volume, surface and depth of the best potential binding site of ClpV1, **(B)** volume, surface and depth of the best potential binding site of ClpV2, and **(C)** volume, surface and depth of the best potential binding site of ClpV3.

Potential binding pockets on the three ClpV proteins were analyzed using the DogSite3 module on the ProteinsPlus server. This was done following confirmation on the difference in the primary and 3D structure of the proteins. Interestingly, the best binding pocket was observed to be around the same area for all three proteins with ClpV1 having the biggest binding pocket (Figure 8). The difference in the size of the binding pockets indicates that ClpV1 may bind bigger proteins or ligands, while ClpV2 and ClpV3 bind to ligands or proteins of similar size. The difference in size between the binding pockets could also be attributed to the presence of the repeat domain (Figure 7).

**FIGURE 9:**
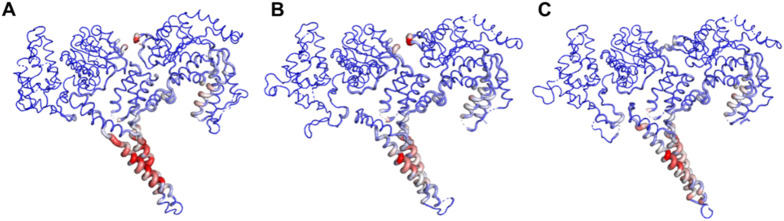
Normal Mode Analysis of ClpV proteins using the DynaMut server. **(A)** ClpV1, **(B)** ClpV2, and **(C)** ClpV3.

Normal Mode Analysis (NMA) was performed to investigate the conformational changes between the ClpV proteins. The color transformations from blue to red in Figure 9 represents the conformational changes and flexibility of the three protein structures (Chen et al., 2025). The most flexible region of the protein appears to be the middle domain with ClpV1 having the most flexible middle region compared to ClpV2 and ClpV3.

Finally, the three ClpV proteins were analyzed using the STRING server to examine their protein-protein interactions (Figure 9). This analysis aimed to explore the similarities and differences in the interacting partners of each ClpV protein. Interestingly, Figure 9 reveals that all three ClpV proteins interact with ClpP, which is unexpected given that ClpV lacks the tripeptide sequence required for ClpP interaction (Schlieker et al., 2005). The mechanism of interaction requires further investigation through *in vitro* studies or additional *in silico* protein-protein binding analyses. However, the observed interaction may result from indirect binding or alternative interaction motifs (Förster et al., 2014).

Our findings indicate that the three ClpV proteins are not structurally identical. Among them, ClpV1 and ClpV2 interact with ClpX, suggesting that the presence of a ClpX-binding site is another distinguishing feature of these proteins. As noted earlier, each strain contains a single copy of ClpX. However, since cells often produce multiple copies of the same protein, ClpX could potentially interact with two different types of ClpV proteins (Alberts et al., 2002). ClpX along with ClpA and ClpB are assumed to play a role in the functioning of the T6SS of *Klebsiella pneumoniae,* however the function of these Clp ATPases in the functioning of this system is yet to be elucidated (Barbosa and Lery, 2019).

### 3.4 MD analysis of three ClpV proteins

**FIGURE 11:**
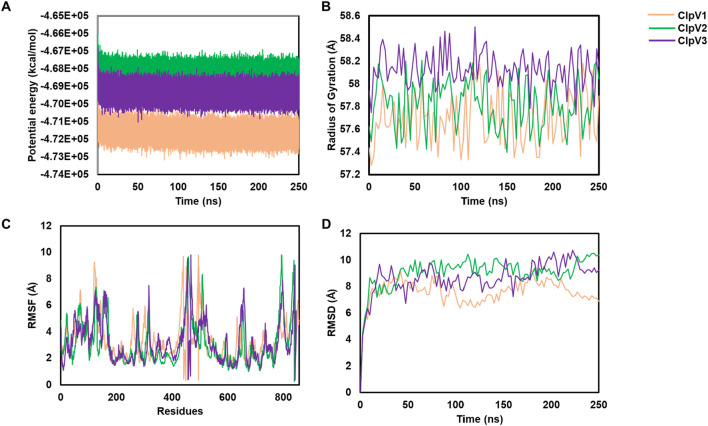
Using potential energy, radius of gyration, RMSF and RMSD to analyze molecular simulations of three ClpV proteins. ClpV1 (pink), ClpV2 (green) and ClpV3 (purple). **(A)** potential energy of alpha carbons observed over 250 ns. **(B)** radius of gyration of alpha carbons observed over 250 ns. **(C)** RMSF for each protein residue. **(D)** RMSD of alpha carbons observed over 250 ns.

MD simulations were performed for the three modelled ClpV proteins. Potential energy, radius of gyration, root mean square deviation (RMSD) and root mean square fluctuation (RMSF) were analyzed to explore the dynamic nature and stability of the proteins (Figure 11). The potential energy profiles of the three proteins remained stable throughout the simulation time of 250 ns (Figure 11A). The radius of gyration (rg) gives an indication of the compactness of the protein, the rg values of all three proteins did not exceed 1 Å therefore indicating that the proteins are compact and stable (Figure 11B). The RMSF shows the residues of the proteins which are the most flexible during the period of simulation (Mohan et al., 2022). The flexibility of all three proteins is significant (>1Å), thus indicating that the proteins are highly dynamic (Figure 11C). The RMSD further confirms the stability of the three proteins, ClpV2 and ClpV3 appear to reach equilibration around 10 ns, while ClpV1 seems to stabilize around 10 ns and continues fluctuating until 100 ns (Figure 11D). The MD run was extended to 400 ns and the dynamic nature of the protein remained consistent with the 250 ns run (data not shown). This along with the RMSF, Rg, and RMSD analysis at 250 ns confirmed that the proteins are stable, and compact. Radial distribution function (RDF) analysis also showed that the proteins had reached full conformational sampling as the systems equilibrated over time (data not shown).

## 4 Discussion

The *Enterobacter* species are a pathogenic group of organisms which evade the action of antibiotics. This makes these pathogens a tremendous burden on the healthcare system and therefore it is important to find alternative targets for drug development to combat their spread. Clp ATPases are a group of proteins which play a role in protein homeostasis, and therefore drugs can essentially be designed to target these proteins and disrupt protein homeostasis. Before drug design, it is important to identify and characterize proteins. In this study, the distribution of Clp ATPases amongst the *Enterobacter* species was investigated to gain an insight into the prevalence of these proteins in the pathogens. Out of the 71 investigated strains it was observed that all contain ClpA, ClpB, ClpX and ClpV, while only 20% contained ClpK (Figure 1). ClpK not being found in all the strains could indicate that a number of the *Enterobacter* strains may not be subjected to heat stress and therefore have not taken up this protein which is easily obtainable through horizontal gene transfer. Additionally, it is possible that there are low numbers of ClpK present due to the presence of ClpB which also plays a role in the survival of bacteria under heat stress (Alam et al., 2021). It was interesting to observe that the pattern of divergence observed between the *Enterobacter* ClpA, ClpB, ClpK and ClpX proteins correlates with the pattern of divergence observed between the *Klebsiella* ClpA, ClpB, ClpK and ClpX proteins (Figure 2) (Motiwala et al., 2022). The ClpV proteins were observed to be genetically variable in comparison to the other Clp ATPase proteins (Figure 2).

The genetic variation observed amongst the investigated ClpV proteins led to further investigation of the proteins. ClpV resets the Type VI Secretion System (T6SS/TssH) by dissembling a sheath which contracts to provide energy for the entry of effector molecules in the cell (Kapitein et al., 2013). The T6SS has been identified to be present in Gram-negative bacteria and plays a role in inducing diarrhea and therefore contributes to the replication of *V. cholera* in the intestine (Kapitein et al., 2013; Förster et al., 2014). Additionally, ClpV has been found to contribute to the pathogenicity and biofilm formation of *P. aeruginosa in vivo* (Förster et al., 2014; Li et al., 2020). The ClpV proteins from *Enterobacter* shared more than 40% similarity with the ClpV proteins from *P. aeruginosa* and *V. cholera* and the proteins lie close to each other on the phylogenetic tree (Figure 3). This suggests that *Enterobacter* ClpV may play a similar role to ClpV from *P. aeruginosa* and *V. cholera,* the ClpV proteins would need to be further investigated through *in vitro* characterization.

Upon observing the variation of ClpV in the *Enterobacter* species it was important to gain an insight into the structural difference of the protein isomers identified in the *Enterobacter* strains. Additionally, it is interesting to note that the one ClpV protein identified from the same strain does not lie next to the other, or on the same branch as the other on the phylogenetic tree (Figure 3). This indicates that despite belonging to the same strain, the ClpV proteins are different to each other (Figure 3). This could be due the presence of distinct T6SS classes in Gram-negative bacteria, each with structural alterations unique to the ClpV protein which binds to them (Förster et al., 2014).

**FIGURE 10:**
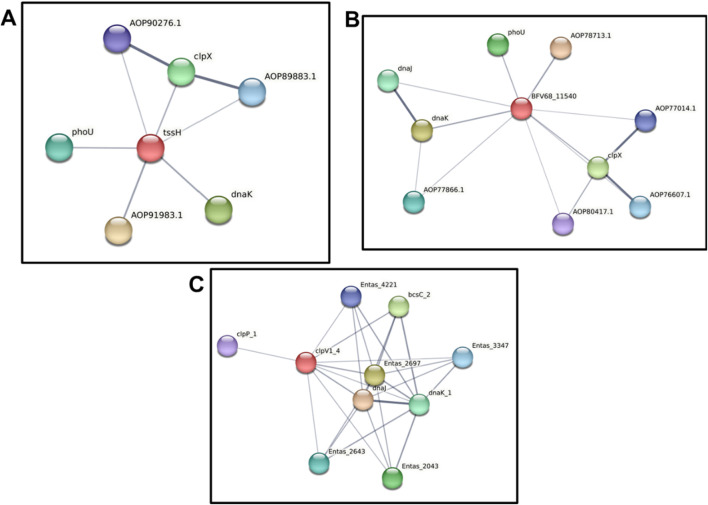
Protein-protein interaction analysis of three ClpV proteins using the STRING server. **(A)** Protein-protein interactions of ClpV1 (red circle), AOP91983.1 is a NAD-dependent epimerase, AOP90276.1 and AOP89883.1 are ATP-dependent Clp protease proteolytic subunits. **(B)** Protein-protein interactions of ClpV2 (red circle), AOP78713.1 is a NAD-dependent epimerase, AOP77866.1 is an antitermination protein, AOP80417.1 is peptidase S14, AOP76607.1 and AOP77014.1 are ATP-dependent Clp protease proteolytic subunits. **(C)** Protein-protein interactions of ClpV3 (red circle), the Entas proteins play a role in the KEGG pathway. The grey lines indicate the interaction confidence, with a thicker line indicating a higher confidence.

Despite being different to each other in terms of sequence, the three ClpV proteins are similar in terms of structure, disorder prediction and physiochemical properties (Figures 4, 5; Table 2). A considerably low percentage of ClpV residues were predicted to be disordered (Figure 5), therefore these proteins can be expressed, purified and used for subsequent biophysical characterisation. Additionally, it was observed that ClpV2 and ClpV3 are more alike in terms of structure, conserved residues, flexibility regions and potential binding sites (Figures 6–9). However, their differences are once again highlighted when analyzing the potential proteins that these ClpV proteins interact with (Figure 10). Furthermore, it was interesting to observe that the most flexible regions were the middle domain of the three proteins, which has been identified as being critical for the disaggregase activity of ClpB and mediates interactions with the Hsp70 chaperone system (Desantis and Shorter, 2012).

The potential energy profile of the three modelled models were investigated and indicated a slight, insignificant shift (Figure 11A), indicating that the models were relatively stable as there was no extreme force experienced by any atom due to the positioning other atoms (Alagu Lakshmi et al., 2020; Hollingsworth and Dror, 2018). Two analyses were used to confirm the dynamic nature of the modelled ClpV proteins. Firstly, the increase in the RMSD values observed over 250 ns indicated confirmational changes (Tiwari and Mohanty, 2013; Buchner, 2019) (Figure 11D). Secondly, the features of the radius of gyration profiles of the ClpV proteins indicated structural transformation, suggesting the constant transformation of the proteins during simulation (Figure 11). This is expected as these proteins are characterised as being chaperones and are thus expected to be highly dynamic in nature (Sučec et al., 2021).

Following the in the preliminary *in silico* investigation of these proteins, future studies could focus on investigating ligands and proteins that interact with these Clp ATPases. Additionally, it would be interesting to investigate whether these proteins all interact with the same Type VI Secretion System (T6SS/TssH). This investigation could allow for the development of potential drugs to target the secretion system-ClpV complex or drugs to target each ClpV homolog could be developed.

## Data Availability

The raw data supporting the conclusions of this article will be made available by the authors, without undue reservation.
